# Role of miR-326 in neonatal hypoxic-ischemic brain damage pathogenesis through targeting of the δ-opioid receptor

**DOI:** 10.1186/s13041-020-00579-4

**Published:** 2020-03-30

**Authors:** Xuan Wang, Han Zhou, Rui Cheng, Xiaoguang Zhou, Xuewen Hou, Jun Chen, Jie Qiu

**Affiliations:** 1 Department of Newborn Infants, Children’s Hospital of Nanjing Medical University, Nanjing, 210008 Jiangsu China; 2 Department of Paediatrics, Nantong First People’s Hospital, Nantong, 226001 Jiangsu China

**Keywords:** Hypoxic-ischemic brain damage, Neonatal infants, miR-326, δ-Opioid receptor, Apoptosis

## Abstract

Hypoxic-ischemic brain damage (HIBD) is a relatively common malignant complication that occurs in newborn infants, but promising therapies remain limited. In this study, we focused on the role of miR-326 and its target gene δ-opioid receptor (DOR) in the pathogenesis of neonatal HIBD. The expression levels of miR-326 and DOR after hypoxic-ischemic injury were examined both in vivo and in vitro. The direct relationship between miR-326 and DOR was confirmed by a dual-luciferase reporter assay. Further, effects of miR-326 on cell viability and apoptosis levels under oxygen glucose deprivation (OGD) were analyzed. The expression levels of miR-326 were significantly lower and DOR levels were significantly higher in the HIBD group than the control group both in vivo and in vitro. Overexpression of miR-326 downregulated the expression of DOR, while suppression of miR-326 upregulated the expression of DOR. The dual-luciferase reporter assay further confirmed that DOR could be directly targeted and regulated by miR-326. MiR-326 knockdown improved cell survival and decreased cell apoptosis by decreasing the expression levels of Caspase-3 and Bax and increasing Bcl-2 expression in PC12 cells after exposure to OGD. Moreover, DOR knockdown rescued the effect of the improved cell survival and suppressed cell apoptosis induced by silencing miR-326. Our findings indicated that inhibition of miR-326 may improve cell survival and decrease cell apoptosis in neonatal HIBD through the target gene DOR.

## Introduction

Hypoxic-ischemic brain damage (HIBD), a relatively common malignant complication, occurs in 1 to 6 of every 1000 live term births and up to 40,000 to 50,000 infants each year in China and is a major cause of neonatal death and neurological dysfunction in infants and children [1, 2]. As reported, approximately 40% of affected infants die in the neonatal period, and approximately 30% of surviving infants have long-term neurological deficits, such as epilepsy, cerebral palsy and cognitive disabilities [3]. Although great efforts have been made, optimal and effective treatments remain lacking, causing HIBD to still be a substantial socioeconomic burden to families and the healthcare system [3]. Therefore, it is urgent to find a potential neuroprotective therapy to improve the prognosis of HIBD infants.

MicroRNAs (miRNAs) are small, noncoding RNAs that negatively regulate gene expression at the posttranscriptional level. It was shown that many miRNAs are expressed in the brain [4], and some of them, such as miR-124, are essential for neuronal development [5]. MiRNAs, including miR-128 [6], miR-210 [7–10], and miR-378 [11], also participate in hypoxic-ischemic (HI) injury. Therefore, miRNAs may be novel therapeutic targets for the treatment of HIBD.

It has been revealed that miR-326 may play a role in neurological disorders; for example, it was downregulated in gliomas and could mediate cell tumorigenicity through Notch signaling [12], while it was upregulated and considered as a diagnostic biomarker in multiple sclerosis [13]. In particular, Kim et al. [14] showed that miR-326 is enriched in cortical neurons. These findings demonstrated the importance of miR-326 in the nervous system. However, the function of miR-326 in neonatal HI brain injury has not yet been studied, which attracted our attention.

MiRNAs play regulatory roles, such as cleavage or translational repression, by targeting mRNAs [15]. We further predicted the target genes of miR-326 using TargetScan and miRNApath, and both analyses found that the delta-opioid receptor (DOR) is one of the miR-326 target genes. DOR is widely distributed in the mammalian central nervous system, especially the cortex and striatum [16]. Notably, DOR is an oxygen-sensitive protein [17] and has neuroprotective effects against hypoxic or ischemic injury [18–20]. Our recent study indicated that DOR plays an important role in protecting against neonatal HIBD by regulating the expression of inflammatory and anti-inflammatory cytokines, which is likely mediated by the Nrf2/HO-1/NQO-1 signaling pathway [21]. Therefore, in this study, we investigated the effect of miR-326/DOR on the pathogenesis of neonatal HIBD. We hope that the findings in our study will provide new therapeutic strategies for neonatal HIBD.

## Methods

### HIBD infants

Ten HIBD infants from the neonatal intensive care unit (NICU) of Children’s Hospital of Nanjing Medical University were selected. Inclusion criteria included (1) term infants with acute fetal distress (prolonged resuscitation need, and/or cord pH < 7.0, and/or Apgar score at 5 min < 5); (2) appearing neurological complications; and (3) clear brain injury diagnosed by magnetic resonance imaging (MRI) or computed tomography (CT). Exclusion criteria included serious brain injuries caused by infection, intracranial hemorrhage, genetic metabolic diseases or others. Ten matched controls were also enrolled. The serum and cerebrospinal fluids (CSF) were collected during the first day after birth and stored at − 80 °C. The research protocol was approved by the hospital medical ethics committee. All parents gave their informed consent.

### HIBD neonatal rats

Pregnant rats (Sprague-Dawley) were obtained from the Laboratory Animal Center of Nanjing Medical University (Nanjing, China) and housed on a 12 h light/dark cycle at 22 °C with free access to food and water. The neonatal HIBD model was established in male rats weighing 18–20 g at postnatal day 7 according to a previously reported method [22]. Briefly, rats were anesthetized with halothane (3.0% for induction, 1.5% for maintenance) in room air, and the left common carotid artery was isolated and ligated with 6–0 surgical silk. The procedure was completed within 10 min. The rectal temperature was controlled by a water blanket placed under the body, and the targeted rectal temperature was maintained at 37 °C during the procedure. To expose rats to hypoxic stress, rats were placed in a plexiglass chamber (30″ W × 20″ D × 20″ H) (BioSpherix, Redfield, NY, USA). The chamber was connected to the outside environment via holes in the wall of the chamber; therefore, CO_2_ levels and humidity in the chamber were kept constant at the ambient levels. O_2_ levels in the chamber were strictly kept at 8 ± 0.5% by constantly flushing with nitrogen that was automatically controlled by a ProOx P110 Oxygen Controller with an E702 Oxygen Sensor (BioSpherix, Redfield, NY, USA). The rats were exposed to hypoxia for 2 h before being returned to their mothers. Rats were placed in a temperature-controlled incubator to maintain the rectal temperature at 37 °C during the whole procedure. The control animals received a sham operation that consisted of left carotid artery exposure without ligation and exposure to hypoxia. Rats were sacrificed by decapitation immediately (0 h) or at 24 h, 48 h and 72 h after HI, and brains were rapidly removed. The cortical tissues of the left hemispheres were dissected on ice, frozen immediately on dry ice, and then stored at − 80 °C until later use. All animal experiments were approved by the Institutional Animal Care and Use Committee of Nanjing Medical University (approval number: IACUC-1902023).

### Assessment of infarct volume

TTC staining was applied to verify the success of HI. In this assay the normal tissue was dyed red by TTC, while the cerebral infarction area was white. At 0 h, 24 h, 48 h and 72 h after HI insult, rats were sacrificed. Brains were quickly removed and cut into 1 mm thick coronal sections on ice. Sections were immersed in 2% TTC solution at 37 °C away from light for 20 min, and the container was shaken slightly every 5 min to fully stain the tissue. Finally, the slices were fixed in 4% paraformaldehyde for imaging.

### Cell culture and oxygen glucose deprivation (OGD)

PC12 rat pheochromocytoma cells were obtained from American Type Culture Collection (Rockville, MD, USA) and cultured in RPMI 1640 culture medium (Life Technologies, MD, USA) supplemented with 10% v/v horse serum (HS), 5% v/v fetal bovine serum (FBS) and appropriate antibiotics in a humidified chamber (5% CO_2_ and 37 °C), all of which were purchased from Invitrogen Life Technologies (Carlsbad, CA, USA).

For the induction of OGD, the medium was switched to RPMI 1640 without glucose after washing the cells twice with glucose-free RPMI 1640. The cells were then placed into an atmosphere of 2% O_2_, 5% CO_2_ and 93% N_2_ at 37 °C for 0 h, 2 h, 6 h or 12 h. Control cells were maintained in glucose-containing RPMI 1640 and incubated in a normoxic incubator for the same time periods.

### Cell transfection

PC12 cells were cultured in a 6-well culture plate (2~4 × 10^5^ cells/well) and maintained overnight in a humidified atmosphere with 5% CO_2_ before transfection. MiR-326 mimics, miR-326 inhibitor, shRNA plasmid against DOR (shDOR) or relative controls (RiboBio, Guangzhou, China) (15 μl) were diluted in RPMI 1640 to a concentration of 400 nM. The solution was mixed with 15 μl Lipofectamine 2000 (Invitrogen, Carlsbad, CA) for a 20 min incubation at room temperature and then added to each well of a 6-well plate (500 μl). The transfection mixture was incubated in a humidified chamber (5% CO_2_ and 37 °C) for 24 h, and then the transfection efficiency was examined by fluorescence microscopy before further assays.

### Quantitative real-time polymerase chain reaction (qRT-PCR)

MiRNAs in serum and CSF were isolated using the miRNeasy Serum/Plasma Kit (Qiagen, Germany). Total RNA from tissues or cells was extracted using TRIzol reagent (Invitrogen, Carlsbad, CA) according to the manufacturer’s instructions. HiScript® II Q Select RT SuperMix for qPCR (Vazyme Biotech, Nanjing, China) was used for reverse transcription of miRNA, and HiScript® II Q RT SuperMix for qPCR (Vazyme Biotech, Nanjing, China) was used for reverse transcription of mRNA following the manufacturer’s instructions. The RT thermal cycle program was as follows: 50 °C for 15 min and 85 °C for 5 s. The qPCR step was performed using a 7900HT Fast Real-Time PCR system with a TaqMan® MicroRNA Assay kit (Applied Biosystems, CA, USA) under the following conditions: 95 °C for 5 min, followed by 40 cycles of 95 °C for 10 s and 60 °C for 30 s. The sequences of the primers are shown in Table 1. The miRNA or mRNA levels were calculated using the 2^-△△CT^ method. All experiments were performed in triplicate.

**Table 1:** Sequences of primer pairs for qRT-PCR

Gene	Forward primer	Reverse primer
has-miR-326	5′-ACTGTCCTTCCCTCTGGGC-3″	5′-AATGGTTGTTCTCCACTCTCTCTC-3’
has-DOR	5′-CAAGATCTGCGTGTTCCT-3′	5′-CGATGACGAAGATGTGGATG-3′
U6	5′-CTCGCTTCGGCAGCACA-3′	5′-TGGTGTCGTGGAGTCG-3′
GAPDH	5′-CATGAGAAGTATGACAACAGCCT-3′	5′- AGTCCTTCCACGATACCAAAGT-3′
rat-miR-326	5′- CGGGCCCTCTGGGCCCTTC-3′	5′- TGGGTTCATTTCTGGGTCTT-3′
rat-DOR	5′-CTGGGACACTGTGACCAAGAT-3’	5′-GTCCAGACGATGACGAAGATG-3’
rat-Bcl-2	5′- GGCATCTTCTCCTTCCAG-3’	5′- CATCCCAGCCTCCGTTAT-3’
rat-Bax	5′- TTTGTTACAGGGTTTCATCCAGG-3’	5′- TTCCAGATGGTGAGCGAGGC − 3’
rat-Caspase-3	5′-GTCTGACTGGAAAGCCGAAACTCT-3’	5′- GAGAAGGACTCAAATTCCGTGGC-3’
Oprd1-RNAi	5′-GATCCCGGCCACCAACATCTACATCTTCTCGAGAAGATGTAGATGTTGGTGGCCTTTTTGGAT-3’	5′-AGCTATCCAAAAAGGCCACCAACATCTACATCTTCTCGAGAAGATGTAGATGTTGGTGGCCGG-3’

has-: the primer sequences of human; rat-: the primer sequences of rat

### Western blot analysis

After washing with ice-cold phosphate-buffered saline (PBS), the tissues or cells were ultrasonically homogenized in RIPA buffer supplemented with protease inhibitor cocktail, and then the homogenates were centrifuged at 12000×g for 15 min at 4 °C. The protein concentration was quantified using a BCA protein assay kit (Pierce, Rockford, IL, USA), and supernatants of homogenates were boiled at 100 °C in laemmli sample buffer (Abcam, Cambridge, MA, UK) for 5 min. Samples were separated on a 10% sodium dodecyl sulfate-polyacrylamide gel and transferred to a polyvinylidene difluoride membrane (Millipore, MA, USA). Membranes were blocked with 5% (m/v) nonfat dry milk in 0.1% Tween 20 (TBS-T; 2 mmol/L Tris-HCl, 50 mmol/L NaCl, pH 7.4) for 2 h at room temperature and subsequently incubated overnight at 4 °C in blocking buffer with anti-β-actin or anti-DOR antibody (Abcam, Cambridge, UK). The membranes were washed with 0.1% Tween 20 and then treated with horseradish peroxidase-conjugated anti-rabbit or anti-mouse IgG for 1 h at room temperature. After washing the membranes three times with TBST, the proteins were visualized with an electrochemiluminescence detection system and quantified by an image analysis system (ImageJ, MD, USA).

### Dual luciferase activity assay

The wild-type and mutated 3′-UTR sequences of DOR (DOR-WT and DOR-MUT, respectively) that were predicted to interact with miR-326 were amplified by PCR and cloned into GV272 (GENECHEM Inc., Shanghai, China). HEK 293 T cells (5 × 10^4^ cells/well) were seeded in a 24-well plate and transfected with DOR-WT, DOR-MUT, miR-326 mimics, and negative control (NC) using Lipofectamine 2000. Cells were harvested after 48 h, and the Dual-Luciferase Reporter Assay System (Promega Corporation, WI, USA) was used to measure the luminous intensity. All experiments were independently repeated three times.

### Cell viability assays

The cell counting kit-8 (CCK-8) assay (Dojindo Molecular Technologies, Tokyo, Japan) was used to test cell viability. PC12 cells in a 96-well plate (5000 cells/well) were transfected with miR-326 mimics, miR-326 inhibitor, miR-326 inhibitor+shDOR and relative negative controls, and OGD was performed following transfection. Cells in 90 μL glucose-free culture medium in each well were mixed with 10 μL CCK-8 solution and incubated for another 1 h in a hypoxic environment. The absorbance was measured at 450 nm using a microplate reader (Thermo Scientific, Vantaa, Finland). All experiments were independently repeated three times.

### Cell apoptosis analysis

PC12 cells in six-well plates (1 × 10^5^ cells/well) were transfected with miR-326 mimics, miR-326 inhibitor, miR-326 inhibitor+shDOR or relative negative controls. After treatment, the cells were harvested by trypsinization and washed twice with ice-cold PBS. Then, cell apoptosis was tested using the annexin V–fluorescein isothiocyanate (FITC) apoptosis detection kit (BD Biosciences, NJ, USA). A FACSCalibur Flow Cytometer (BD Biosciences, NJ, USA) was used to distinguish apoptotic cells, and the results were analyzed by FlowJo software (Tree Star Corp, Ashland, OR). All experiments were independently repeated three times.

### Statistical analysis

All values are expressed as the mean ± SEM. Student’s paired *t*-test was used to compare values between two groups. Statistical analysis was performed using SPSS statistical software package v22.0 (SPSS, Chicago, IL, USA). Statistical significance was determined based on *P* < 0.05.

## Results

### The expression levels of miR-326 and DOR under HI conditions in vivo and vitro

**Fig. 1:**
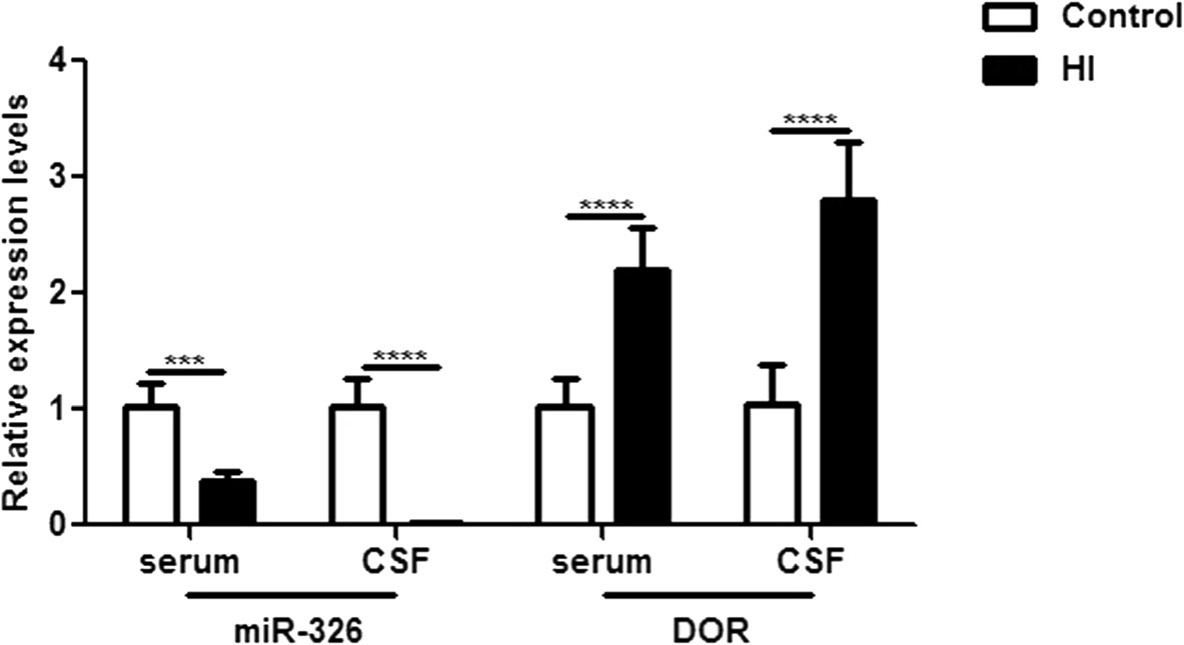
The expression levels of miR-326 and DOR in serum and cerebrospinal fluids of HIBD infants. Both in serum and CSF, the expression of miR-326 was significantly lower in HIBD infants than in controls (^***^P < 0.005; ^****^P < 0.001), while the expression of DOR was significantly increased in HIBD infants (^****^P < 0.001)

We examined the expression levels of miR-326 and DOR under HI conditions in vivo and in vitro. First, we measured the levels in the serum and CSF of HIBD infants using qRT-PCR. Our results showed that the expression of miR-326 in both the serum and CSF of HIBD infants was significantly lower than that in controls. In contrast, the expression of DOR was significantly increased in infants with HIBD (Fig. 1).

**Fig. 2:**
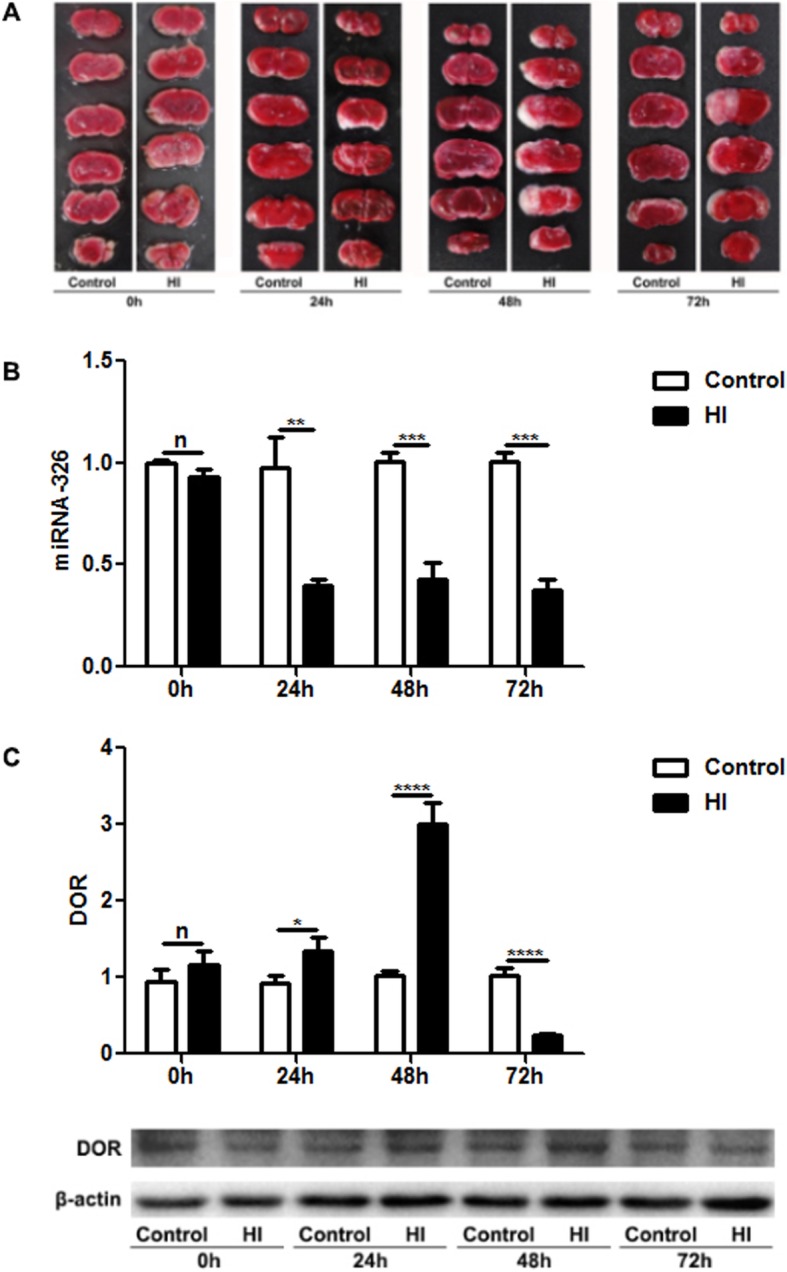
The expression levels of miR-326 and DOR in cortical tissues of neonatal rats with HIBD. (**a**) The HIBD rat model was verified by TTC staining. There were obvious white infarctions in the left cerebral cortex in the HI group, while the right cerebral cortex under HI and both sides of the control group did not show any infarction areas. (**b**) The expression levels of miR-326 did not change immediately at 0 h after HI (^n^*P* > 0.05) and significantly decreased at 24 h, 48 h and 72 h after HI injury in neonatal rats (^**^P < 0.01; ^***^P < 0.005). (**c**) The mRNA expression levels of DOR were greatly upregulated at 24 h and 48 h after HI injury (^*^P < 0.05; ^****^P < 0.001) but were significantly downregulated 72 h after HI injury (^****^P < 0.001). The protein expression levels of DOR were consistent with the mRNA expression levels

Then, we studied the expression levels of miR-326 and DOR using cortical tissues of neonatal rats with HIBD. The HIBD rat model was verified by TTC staining, and the results showed obvious infarction in the left cerebral cortex in the HI group compared with the right cerebral cortex of the HI group and both sides of the controls (Fig. 2a). As shown in Fig. 2b and c, the expression levels of miR-326 and DOR did not change immediately at 0 h after HI injury (*P* > 0.05). Then, the levels of miR-326 were significantly downregulated at 24 h, 48 h and 72 h (*P* < 0.05; Fig. 2b). Interestingly, the mRNA and protein expression levels of DOR were significantly upregulated at 24 h and 48 h but downregulated at 72 h after HI injury (*P* < 0.05; Fig. 2c).

**Fig. 3:**
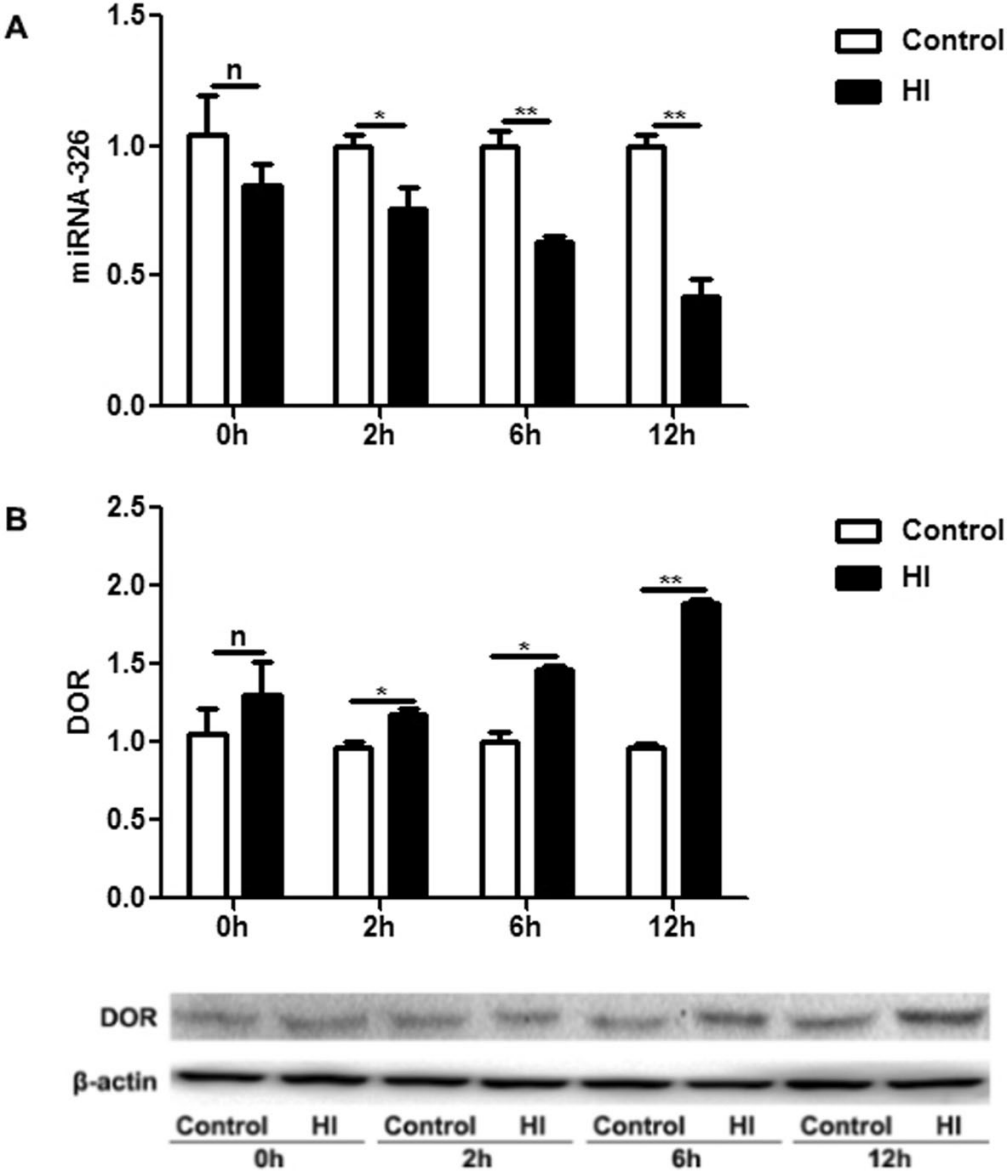
The expression levels of miR-326 and DOR in PC12 cells under OGD. (**a**) The expression levels of miR-326 did not change immediately in PC12 cells at 0 h after OGD (^n^*P* > 0.05) and then were significantly downregulated at 2 h, 6 h and 12 h after OGD (^*^P < 0.05; ^**^P < 0.01). (**b**) The mRNA and protein expression levels of DOR did not change immediately after OGD (^n^*P* > 0.05) and were then upregulated at 2 h, 6 h and 12 h after OGD (^*^P < 0.05; ^**^P < 0.01)

The results of cell culture experiments are shown in Fig. 3. Consistent with the results in humans and animals, the mRNA expression levels of miR-326 and DOR did not change immediately in PC12 cells at 0 h after OGD (*P* > 0.05). The expression levels of miR-326 were significantly downregulated, and DOR were significantly upregulated in PC12 cells at 2 h, 6 h, and 12 h after OGD (*P* < 0.05). The protein expression levels of DOR confirmed the qRT-PCR results.

### DOR is the target gene of miR-326

**Fig. 4:**
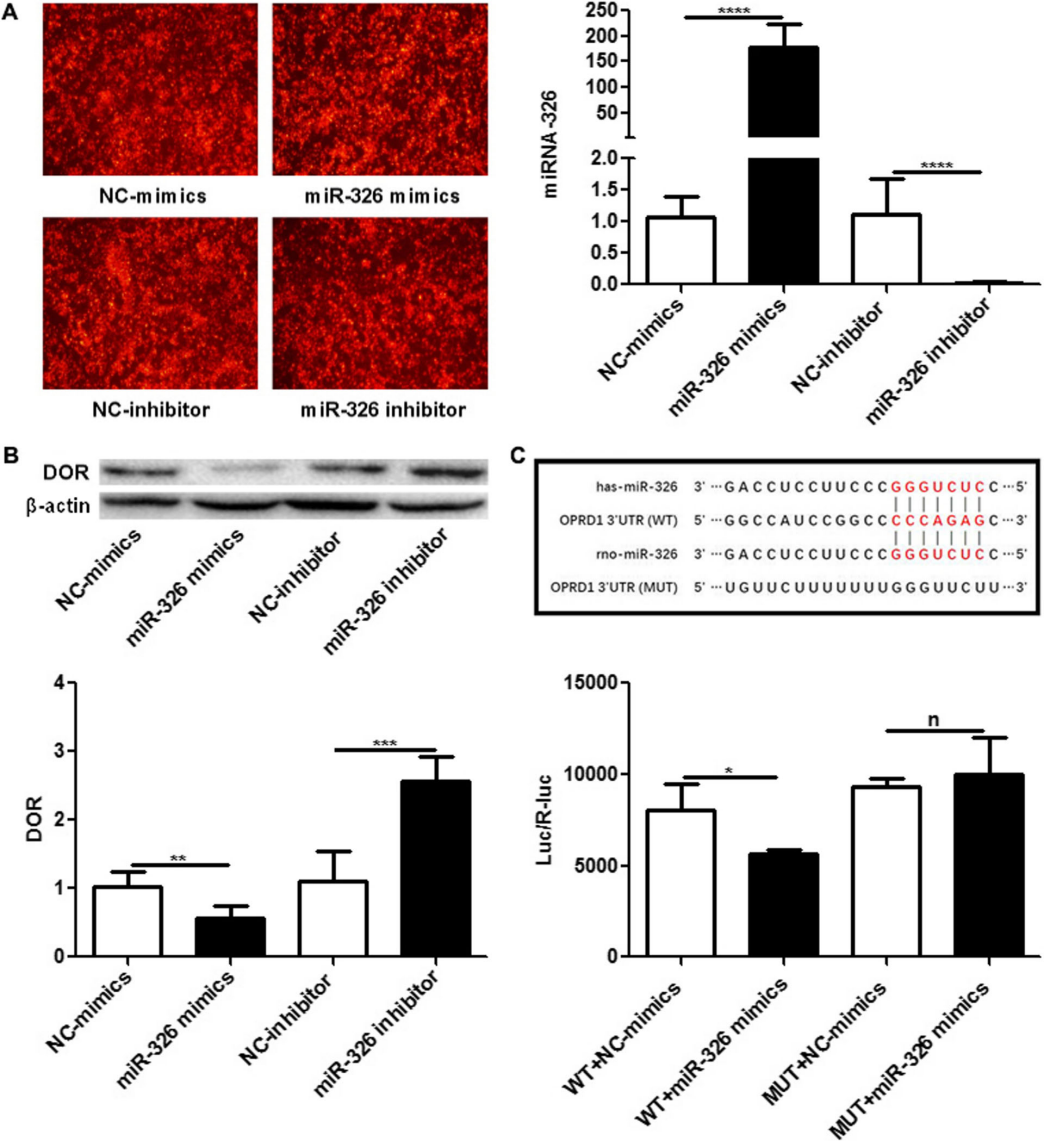
DOR is the target gene of miR-326. (**a**) The transfection efficiencies of PC12 cells transfected with miR-326 mimics, miR-326 inhibitor and relative controls, as represented by red fluorescence, were approximately 80–90% after transfection for 24 h. The expression levels of miRNA-326 were significantly upregulated in PC12 cells transfected with miR-326 mimics and significantly downregulated in PC12 cells transfected with miR-326 inhibitor compared with those in the relative controls (^****^P < 0.001). (**b**) The mRNA and protein expression levels of DOR were significantly downregulated in PC12 cells transfected with miR-326 mimics (^**^P < 0.01) and significantly upregulated in PC12 cells transfected with miR-326 inhibitor compared with those in the controls (^***^P < 0.005). (**c**) Schematic representation of the predicted miR-326-binding site in the DOR 3′-UTR in both humans and rats. The luciferase activity of cells transfected with the DOR-wild-type (WT) reporter, which contained the predicted miR-326-binding site with the target sequence of the wild-type 3′-UTR, was significantly suppressed by cotransfection with miR-326 mimics (^*^P < 0.05), while there was no significant difference in cells transfected with the corresponding DOR-mutated-type (MUT), which contained a putative miR-326 binding site with a mutant region in the 3′-UTR (^n^*P* > 0.05)

We transfected PC12 cells with miR-326 mimics, miR-326 inhibitor, or the relative controls. The transfection efficiency, which was represented by red fluorescence, was approximately 80–90% after transfection (Fig. 4a). Furthermore, the expression of miR-326 was significantly increased after transfection with miR-326 mimics and significantly decreased after transfection with miR-326 inhibitor (*P* < 0.05; Fig. 4a). These results revealed that the transfection was effective.

Then, we detected the mRNA and protein expression of DOR in transfected PC12 cells under OGD. The results showed that the overexpression of miR-326 significantly reduced the expression of DOR, whereas the suppression of miR-326 significantly increased DOR expression (*P* < 0.05; Fig. 4b).

A luciferase assay was performed to further confirm that miR-326 directly targets DOR. The luciferase activity of DOR-wild-type (WT) cells was significantly suppressed by cotransfection with miR-326 mimics (*P* < 0.05), while there was no change in DOR-mutated-type (MUT) cells (*P* > 0.05; Fig. 4c), indicating that DOR is the target of miR-326.

### MiR-326 affects cell viability and apoptosis in vitro by directly targeting DOR

**Fig. 5:**
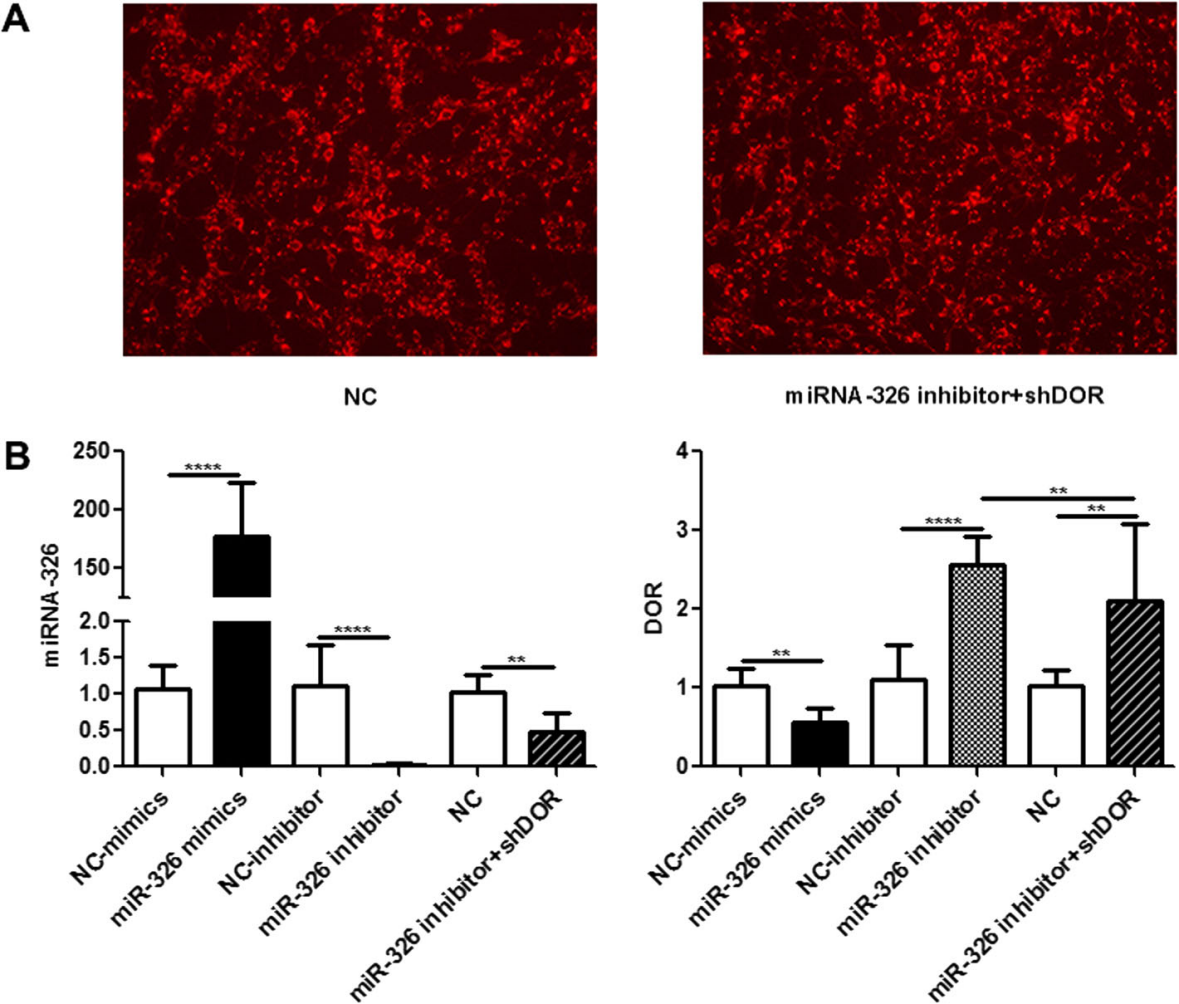
Transfection efficiency of transfected cells. (**a**) The transfection efficiencies of PC12 cells transfected with both miR-326 inhibitor and shDOR or the relative control (NC) were 80–90%. (**b**) After cotransfection with miR-326 and DOR inhibitors at the same time, the expression levels of miR-326 were decreased, and DOR expression was higher than that in the control, although lower than that in cells transfected with miR-326 inhibitor (^**^P < 0.01; ^****^P < 0.001)

The transfection efficiency of PC12 cells transfected with both miR-326 inhibitor and shDOR or the relative control was 80–90%, as shown in Fig. 5a. After suppressing the expression of miR-326 and its target gene DOR at the same time, we found that miR-326 was decreased and DOR expression was higher than that in the control, although lower than that in cells transfected with only miR-326 inhibitor (*P* < 0.05; Fig. 5b).

**Fig. 6:**
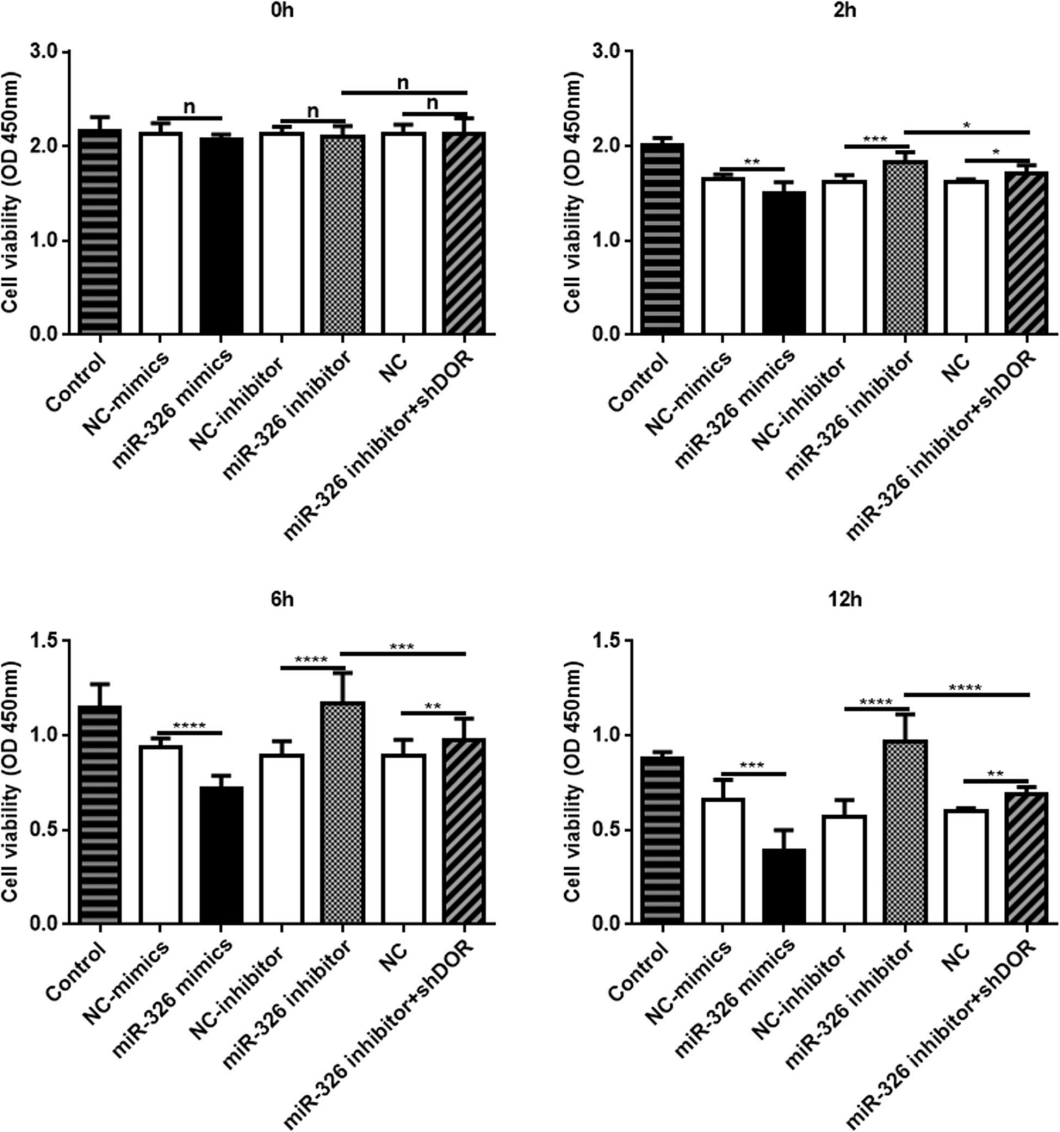
Effects of miRNA-326 on cell viability under OGD. The viability of PC12 cells without transfection (Control) gradually declined after OGD. Cell viability did not differ between the other groups and their controls at 0 h after OGD (^n^*P* > 0.05). The cell survival rate was significantly decreased in PC12 cells transfected with miR-326 mimics and was significantly increased in PC12 cells transfected with miR-326 inhibitor at 2 h, 6 h and 12 h after OGD (^**^P < 0.01; ^***^P < 0.005; ^****^P < 0.001). The survival rate showed a downward tendency after cotransfection of miR-326 and DOR inhibitors compared to miR-326 inhibitor only (^*^P < 0.05; ^***^P < 0.005; ^****^P < 0.001)

We then analyzed the viability and apoptosis levels of PC12 cells after transfection with miR-326 mimics or miR-326 inhibitor or cotransfection of miR-326 inhibitor and shRNA plasmid targeting DOR. As indicated in Fig. 6, cell viability did not change immediately at 0 h after OGD (*P* > 0.05). Then, overexpression of miR-326 reduced cell survival, and suppression of miR-326 increased cell survival at 2 h, 6 h and 12 h after OGD (*P* < 0.05). The survival rate showed a downward tendency after cotransfection of miR-326 and DOR inhibitors compared to miR-326 inhibitor only (*P* < 0.05).

**Fig. 7:**
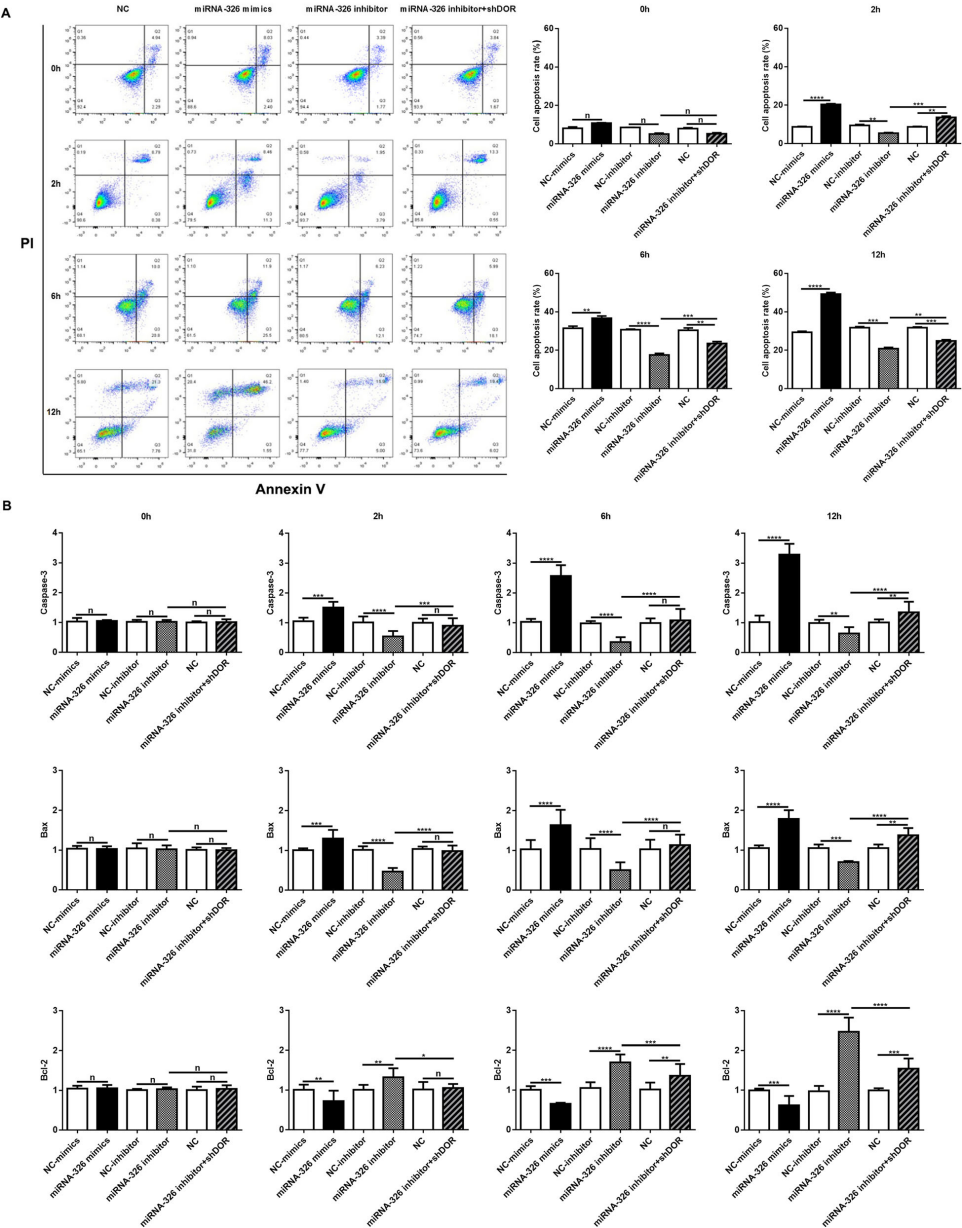
Effects of miRNA-326 on cell apoptosis under OGD. (**a**) The cell apoptosis rate did not change immediately at 0 h after OGD (^n^*P* > 0.05). Apoptosis was significantly increased in PC12 cells transfected with miR-326 mimics and was significantly decreased in PC12 cells transfected with miR-326 inhibitor at 2 h, 6 h and 12 h after OGD (^**^P < 0.01; ^***^P < 0.005; ^****^P < 0.001). In PC12 cells cotransfected with shDOR and miR-326 inhibitor, the cell apoptosis rate that resulted from the miR-326 inhibitor was partially rescued (^**^P < 0.01; ^***^P < 0.005). (**b**) Overexpression of miR-326 increased the expression levels of Caspase-3 and Bax and decreased Bcl-2 at 2 h, 6 h and 12 h after OGD but not at 0 h (^n^*P* > 0.05; ^**^P < 0.01; ^***^P < 0.005; ^****^P < 0.001). Inhibition of miR-326 had opposite effects, which were rescued by cotransfection of miR-326 and DOR inhibitors (^*^P < 0.05; ^**^P < 0.01; ^***^P < 0.005; ^****^P < 0.001)

Flow cytometry assays demonstrated that overexpression of miR-326 increased the cell apoptosis rate, whereas suppression of miR-326 decreased cell apoptosis at 2 h, 6 h and 12 h after OGD. However, when we cotransfected miR-326 and DOR inhibitors, the effect of the miR-326 inhibitor was partially rescued (*P* < 0.05; Fig. 7a). Furthermore, overexpression of miR-326 increased the expression levels of Caspase-3 and Bax and decreased Bcl-2 at 2 h, 6 h and 12 h after OGD compared with the control. Inhibition of miR-326 had the opposite effects, which were rescued by cotransfection of miR-326 and DOR inhibitors (*P* < 0.05; Fig. 7b).

## Discussion

As a common health problem of neonatal infants, HIBD leads to many neurological deficits. Extensive resources are required for the treatment and care of HIBD sequelae. However, there is still a lack of optimal neuroprotective therapies against HIBD. MiRNAs, which are highly conserved non-protein-coding RNA molecules, regulate approximately 60% of human genes [23] and play critical roles in a variety of biological processes by targeting mRNAs [24]. In recent years, researchers have realized the significance of miRNAs in human diseases, including HI injury [25, 26]. Although miR-326 was reported to perform important roles in cancerous, autoimmune, autoinflammatory and other pathological conditions, there is no evidence that it has functions in neonatal HIBD [26, 27]. In the current study, we first demonstrated the potential role of miR-326 in neonatal HIBD.

We used the HI model in humans (Fig. 1), rats (Fig. 2) and cells (Fig. 3) to study the expression levels of miR-326 and DOR. Overall, the expression of miR-326 was decreased and DOR was increased under HI injury. The negative correlation between the expression levels of miR-326 and DOR in HI injuries and the results of the luciferase reporter assay proved our hypothesis that DOR is a target gene of miR-326 (Fig. 4). DOR has been reported to be neuroprotective and actively participates in the control of neuronal survival in HI insults [28, 29]. Therefore, we inferred that downregulation of miR-326 might upregulate the expression of DOR and then exert a neuroprotective function in neonatal HIBD.

What attracted our attention is that DOR levels declined 72 h after HI injury in neonatal rats (Fig. 2), while the expression of miR-326 was also decreased. We speculated that HI damage gradually aggravated cell injury over time and resulted in the breakdown of self-regulation and that the decrease in miR-326 could not upregulate DOR to protect against HI damage. This demonstrated that long-term injury might lead to irreparable damage, and it is urgent to start treatment as soon as possible after HI brain injury in infants.

After cotransfecting inhibitors of miR-326 and DOR, we noted that the expression of miR-326 was decreased while the expression of DOR was still higher than that in the control (Fig. 5). This finding further supported the notion that the downregulation of miR-326 could significantly upregulate the level of DOR, although it was decreased by the DOR inhibitor.

It is well known that apoptosis is programmed cell death and plays a role in physiological homeostasis, growth and development. Doycheva et al. [30] demonstrated the occurrence of apoptosis following HIBD in neonatal rats. Zhao et al. [31] also showed that HIBD could be improved by suppressing neural apoptosis. Previous studies demonstrated that overexpression of miR-326 decreased cell viability, inhibited cell growth [12, 32] and increased cell apoptosis [33, 34]. However, there is no current evidence about the role of miR-326 in cell viability and apoptosis after HI injury. Our results showed that overexpression of miR-326 resulted in a significant decrease in cell survival (Fig. 6) and increase in cell apoptosis rate (Fig. 7), while suppression of miR-326 had the opposite result and was partially rescued by cotransfection of miR-326 and DOR inhibitors. Therefore, we presumed that miR-326 suppression could protect neurocytes from OGD-induced neuronal death by targeting DOR. However, although OGD downregulates the expression of miR-326 which decreases cell apoptosis, the cell viability gradually declined after OGD as shown in the control group of Fig. 6. We speculated that the declined expression of miR-326 after OGD may be a feedback to HI damage and play a protective role through increasing DOR, which may be a protective mechanism suffering HI injuries. Some miRNAs also had similar effects under HI injuries, for example, miR-21 expression was significantly up-regulated after stroke [35] but its overexpression had a protective effect on ischemia-induced cell apoptosis [36]. Nevertheless, the pathogenesis of HIBD is a complex progress which includes numerous genes and mechanisms. MiR-326 is just one of genes and apoptosis is just one of complex mechanisms. OGD, which mimics the effects of HI injury in vitro, influences cell growth and lead to a decrease in cell viability [37, 38], which is aggravated along with an increased OGD time [39]. Without effective interventions, cell damage would be unavoidable following long time OGD. Therefore, at last, the cellular viability of the normal control group must be declined after OGD even though the downregulation of miR-326 had a protective role against HI injuries.

It has also been reported that miR-326 could regulate proapoptotic factors [32], and DOR activation protected cells from hypoxia by downregulating cleaved Caspase-3, while its inhibition induced the opposite effect [40]. Therefore, we studied the effects of miR-326 and DOR on cell apoptotic factors under HI injury. Our results showed that overexpression of miR-326 decreased the expression of DOR, leading to apoptosis of PC12 cells after OGD by increasing Caspase-3 and Bax and decreasing Bcl-2. In contrast, inhibition of miR-326 had the opposite effects, while cotransfection of miR-326 and DOR inhibitors rescued these effects. Therefore, it could be concluded that inhibition of miR-326 plays a positive role in neonatal HIBD by upregulating the expression of DOR by decreasing Caspase-3 and Bax and increasing Bcl-2. For the first time, we demonstrated that it is crucial to improve the efficacy of neonatal HIBD therapies via miR-326 and DOR.

**Fig. 8:**
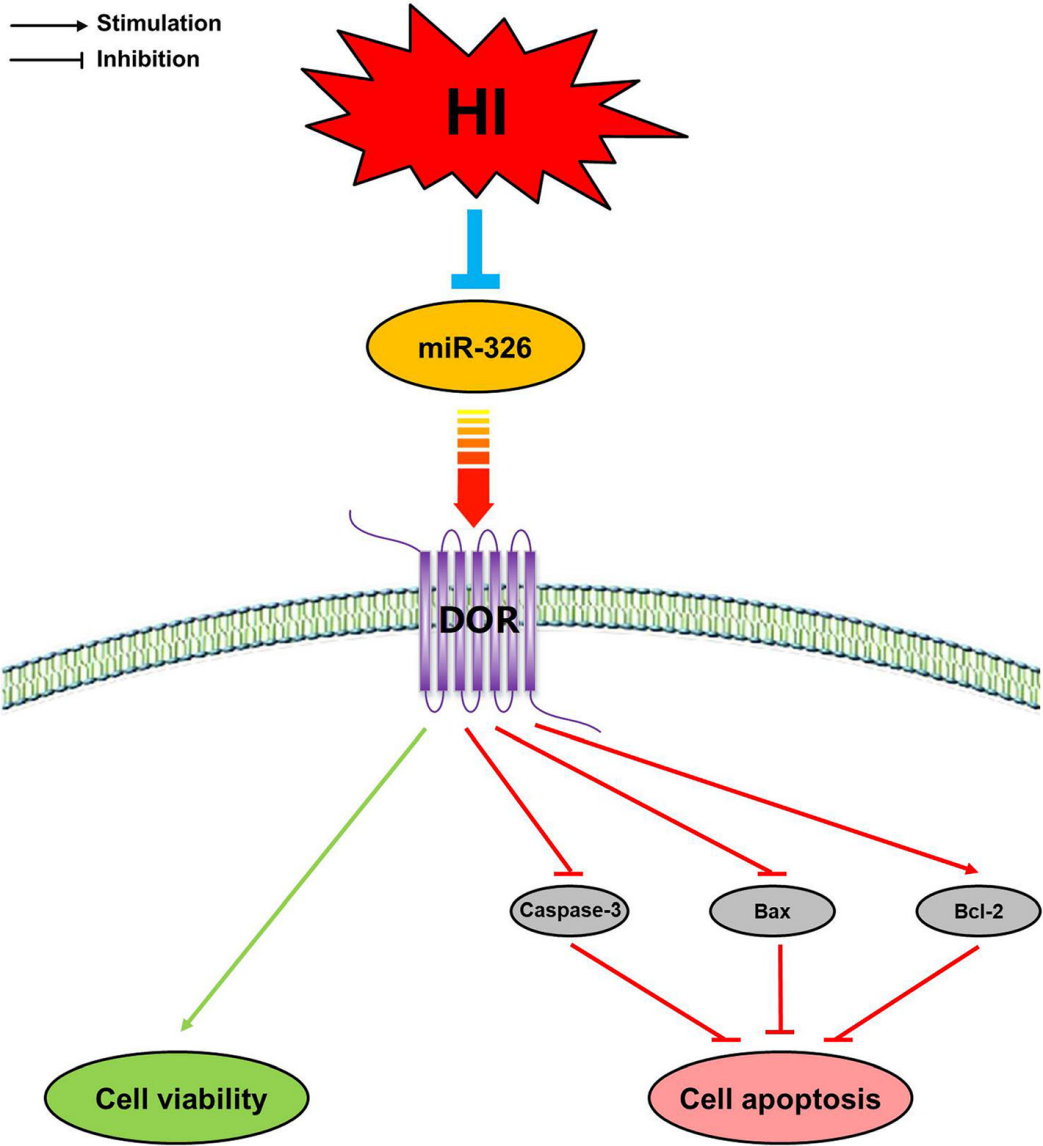
Diagrammatic Drawing. HI insult downregulated the expression level of miR-326 and upregulated the expression level of DOR, which is the direct target of miR-326. The inhibition of miR-326 improved cell survival and decreased cell apoptosis by targeting DOR in neonatal HIBD

In conclusion, our results showed that HI insult downregulated the expression of miR-326 and upregulated the expression of its target gene DOR. Inhibition of miR-326 could improve cell survival and suppress cell apoptosis through the direct targeting of DOR in neonatal HIBD (Fig. 8). Our study provided preliminary findings about the potential role of miR-326 and its target gene DOR in the pathogenesis of neonatal HIBD, and more studies are needed to elucidate further potential mechanisms.

## Data Availability

The datasets generated and analyzed during the current study are not publicly available due to privacy concerns but are available from the corresponding author upon reasonable request.

## Abbreviations

CCK-8: Cell counting kit-8
CSF: Cerebrospinal fluids
CT: Computed tomography
DOR: δ-opioid receptor
FBS: Fetal bovine serum
HI: Hypoxic-ischemic
HIBD: Hypoxic-ischemic brain damage
HS: Horse serum
miRNAs: microRNAs
MRI: Magnetic resonance imaging
NC: Negative control
NICU: Neonatal intensive care unit
OGD: Oxygen-glucose deprivation
PBS: Phosphate buffer saline
qRT-PCR: Quantitative real-time polymerase chain reaction
shDOR: shRNA plasmid of DOR
